# Controlling Crystal Morphology of Anisotropic Zeolites with Elemental Composition

**DOI:** 10.1021/acs.cgd.3c01312

**Published:** 2024-03-08

**Authors:** Ondřej Veselý, Mariya Shamzhy, Wiesław J. Roth, Russell E. Morris, Jiří Čejka

**Affiliations:** † Faculty of Sciences, Charles University, Hlavova 8, 128 43 Prague 2, Czech Republic; ‡ Faculty of Chemistry, Jagiellonian University, Gronostajowa 2, 30-387 Krakow, Poland; § EaStChem School of Chemistry, University of St. Andrews, North Haugh, Fife, St. Andrews KY16 9ST, U.K.

## Abstract

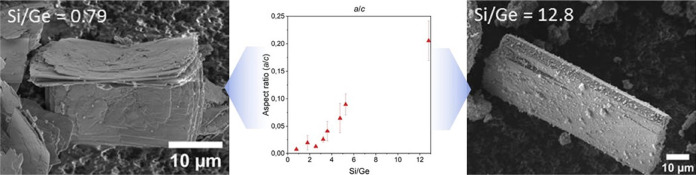

The morphology of zeolite crystals strongly affects their textural, catalytic, and mechanical attributes. However, controlling zeolite crystal morphology without using modifiers or structure-directing agents remains a challenging task because of our limited understanding of the relationships between zeolite crystal shape, crystallization mechanism, and composition of the starting synthesis mixture. In this study, we aimed at developing a general method for controlling the morphology of zeolites by assessing the impact of the Si/T molar ratio of the synthesis gel on the growth rate of zeolite crystals in various crystallographic directions and on the final crystal morphology of the **UTL** germanosilicate with a 2D system of intersecting 14- and 12-ring pores. Our results showed that flat **UTL** crystals progressively thicken with the Si/Ge molar ratio, demonstrating that Ge concentration controls the relative rate of crystal growth in the perpendicular direction to the pore system. The morphology of other zeolites and zeotypes with an anisotropic structure, including **AFI** (12R), **IFR** (12R), **MWW** (10–10R), and **IWW** (12–10–8R), can also be predicted based on their Si/T ratio, suggesting a systematic pattern across zeolite structures and in a wide range of zeolite framework elements. Combined, these findings introduce a facile and cost-efficient method for directly controlling crystal morphology of zeolites with anisotropic structures with a high potential for scale-up while providing further insights into the role of elemental composition in zeolite crystal growth.

## Abstract 2

This study explores how varying Si/Ge ratios in zeolite synthesis gels affect crystal growth direction and morphology. Results offer a method to control zeolite crystal morphology without harmful additives.

## Introduction

Silicate and aluminosilicate zeolites stand out for their role in numerous industrial processes involving adsorption, gas separation, and shape-selective acid catalysis.^1−9^ Related frameworks based on aluminophosphates and including isomorphous substitution with Ti, Fe, and other elements are also applied in the industry.^10^ Zeolites are microporous crystalline materials with frameworks consisting of corner-sharing, four-connected TO_4_ (T = Si, Al, Ge, and Fe, among others) tetrahedra.^1,11,12^ They have a wide range of applications thanks to their thermal stability, safe handling, and activity versatility/tuneability through changes in structure, porosity, and composition. Their textural (e.g., pore size, pore accessibility, and surface area), acid (e.g., acid strength and concentration of acid sites), and physical (e.g., particle size and mechanical strength) properties, in particular, vary with structure, elemental composition, and crystal size and morphology.^8,13−17^

The morphology of zeolites can be adjusted using growth modifiers or specially designed structure-directing agents (SDAs).^18−23^ However, these agents significantly increase synthesis costs and hinder potential large-scale applications, even though the main criterion is usually clear-cut performance benefits. Moreover, industrial production demands a simple and general method for controlling zeolite morphology by altering fundamental synthesis conditions (e.g., temperature, stirring, water content, or elemental composition of the synthesis gel).^24^

Synthesis conditions can be changed to control zeolite crystal growth and morphology, as previously illustrated with **LTL** or **FAU** zeolites.^22,25−27^ However, this approach is only valid for specific morphologies, lacking general principles applicable to a wide set of zeolite frameworks. The difficulty in setting guidelines derives from our limited understanding of zeolite growth mechanisms and from the lack of clear-cut relationships between synthesis conditions (e.g., gel composition), crystallization rate, and final crystal morphology.

Zeolite crystal morphology arises from the equilibrium between crystal growth rates in all crystallographic directions as equal relative crystal growth rates produce highly symmetric crystals (e.g., cubes, octahedra), while markedly different rates result in less isotropic crystal shapes.^28^ In this context, the condensation of silicate species from the synthesis gel into a complete cage or a building unit with as few uncondensed silanol groups as possible has been proposed as the rate-determining step of zeolite crystal growth.^29^ Despite extensively describing zeolite growth mechanisms on pure silica zeolite models and validating their conclusions by comparing predicted crystal morphologies to experimentally prepared zeolite crystals, these modeling studies overlooked the effects of synthesis conditions, such as elemental composition, choice of SDA, temperature, and water content, on zeolite crystal morphology due to the computational requirements of such modeling.^30,31^ For these reasons, controlling the crystal morphology of zeolites remains a challenging task.

In this study, we aimed at developing a method for controlling the morphology of zeolites by assessing the effect of heteroelement (i.e., nonsilicon) framework atoms on the crystal morphology of a wide range of zeolites with varying topologies and elemental composition. For our experiments, we chose germanosilicate zeolites **UTL** (14–12R) and **IWW** (12–10–8R). **UTL** forms flat rectangular crystals with a two-dimensional channel system running parallel to the largest plane. Accordingly, we hypothesized that the anisotropy of its pore system or the uneven size of the building units would affect crystal growth rates along different crystallographic axes and that the growth rate along the *a*-axis should vary with the Si/Ge ratio, given the presence of Ge-rich D4R units in this direction. **IWW** germanosilicate contains a three-dimensional channel system composed of 12–10- and 8-rings and is thus suitable for determining whether the morphology evolution originates from the position of the Ge-rich D4Rs, the direction of the pores, or the size of the building units. By scanning electron microscopy (SEM), we examined the crystal morphology of **UTL** and **IWW** germanosilicates and their variation as a function of the Si/Ge molar ratio. Our findings provide a general and efficient approach to controlling zeolite crystal morphology by changing the Si/T ratio of the synthesis gel.

## Results

### **UTL** Zeolite

**Figure 1:**
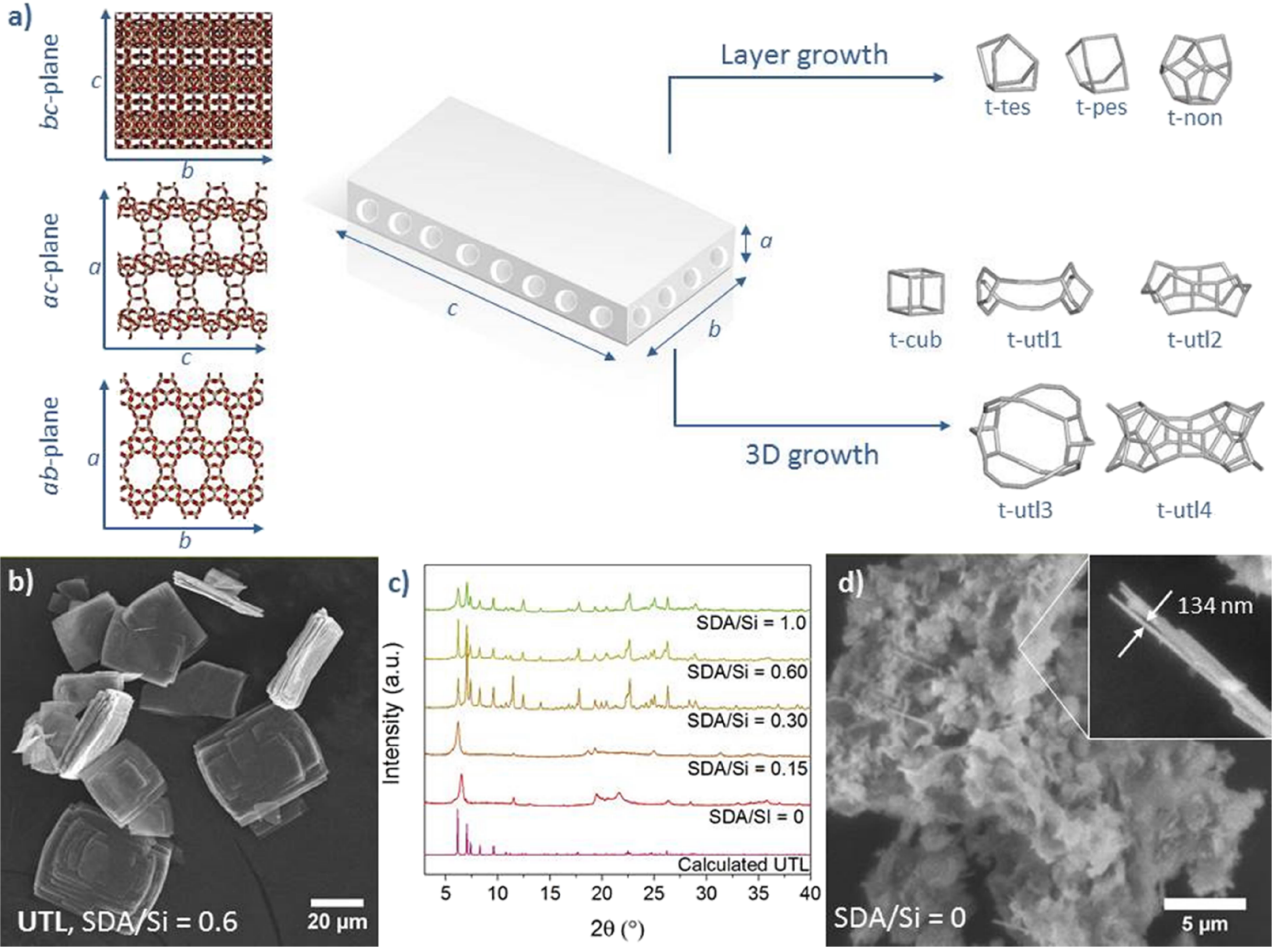
(a) Schematic representation of a **UTL** zeolite crystal with projections along the *a*-, *b*-, and *c*-axes and building units involved in layer and 3D growth; (b) SEM image of **UTL** (Si/Ge = 3.22; SDA/Si = 0.6); (c) powder XRD patterns of **UTL** synthesized with various SDA/Si ratios; and (d) SEM image of a material prepared under SDA/Si = 0.

The initial experiments were performed with **UTL** germanosilicate (IM-12,^32^ ITQ-15^33^) for its structural features. More specifically, the **UTL** topology contains intersecting 14R and 12R channels arranged into a 2D pore system. These channels are sandwiched between parallel nonporous *pcr*^1^ layers connected by cubical D4R units into the three-dimensional **UTL** framework (see Figure 1a).^32^ Furthermore, **UTL** zeolite often crystallizes as a germanosilicate with germanium atoms preferentially located in D4R units, while the layers are primarily composed of silicon as T atoms.^35−37^ As such, the **UTL** zeolite should provide us with a convenient tool for studying the variation of morphology as a function of synthesis conditions.

**UTL** crystals resemble thin flat platelets with dimensions ranging from 0.5 to 1.0 μm along the *a*-axis (thickness) and from 10 to 50 μm in *b-* and *c-*axes, as reported in previous studies.^32,38−40^ However, whether the crystal size strongly depends on synthesis conditions remains unclear. For this reason, we minimized sources of variability by using the same equipment (20 mL Teflon-lined steel autoclaves and rotary oven), settings (rotation speed, and heating rate, among other parameters), and conditions (synthesis temperature and time) in our experiments. We also ensured that each synthesis was carried out under the same initial pH by adjusting its value to pH = 10.5 prior to the hydrothermal treatment.

Under these synthesis conditions, we prepared **UTL** zeolite with a framework Si/Ge molar ratio of 3.22 and crystals with an average size of 43.7 × 32.5 × 0.84 μm^3^ (Figure 1b). The crystals consisted of flat rectangular lamellae stacked and intergrown into larger crystals. The crystal lamellae were rectangular, with 90, 90, and 105.1° angles between the facets, matching the angles of the **UTL** unit cell and, thus, enabling us to easily identify the crystallographic axes. The crystallographic axes were assigned for each subsequent sample individually (Figure S1 illustrates the axis assignment on UTL with Si/Ge = 5.26). This flat crystal morphology derives from differences in the relative rate of crystal growth caused by the structural features of the **UTL** framework (vide supra).^28^

For clarity, we analyzed layer growth (*b-* and *c-*axis) and growth in the third dimension (*a*-axis) separately. **UTL** zeolite layers are propagated through the formation of small *t-tes*, *t-pes,* and *t-non* cages. In contrast, the growth along the *a*-axis involves not only the rapid formation of D4R (*t-cub*) units but also the slow formation of large cages at the channel intersections (i.e., *t-utl-1*, *t-utl-2*, *t-utl-3*, or *t-utl-4*). As a result, layer propagation is faster than growth in the third dimension, thereby explaining the flat, wide crystals.

Alternatively, we may interpret the large cages as a byproduct of the subsequent formation of *t-cub*, *t-non*, and *t-tes* or *t-pes* cages into an “arch”, which corresponds to the *pcr* layer. However, even from this point of view, the *a*-axis growth requires multiple steps, involving a large number of less stable and partly condensed Si species. The formation of these large cages also requires stabilization by SDA molecules, as shown by repeating the **UTL** synthesis with a lower SDA concentration in the synthesis mixture. Synthesis with SDA/Si ≥ 0.3 yielded a fully crystalline **UTL** zeolite (Figure 1c), while synthesis with SDA/Si ≤ 0.15 yielded only a partly ordered lamellar material with 134 nm-thick sheets (Figure 1d). The results confirm that an SDA content above SDA/Si = 0.3 is crucial for stabilizing the large cavities and forming the **UTL** framework.

**Figure 2:**
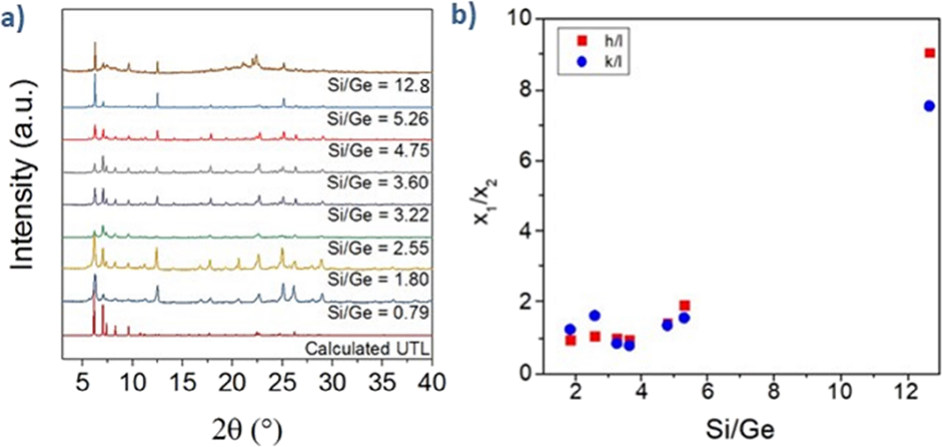
(a) Powder XRD patterns of **UTL** samples; (b) variation of the *h*/*l* and *k*/*l* aspect ratios of the crystallites.

We tested our first hypothesis by synthesizing a series of **UTL** zeolites with Si/Ge ratios ranging from 0.79 to 12.8. The synthesis mixtures possessed the same pH as well as SDA/Si molar ratios. We did not consider changes in local SDA concentration or in the strength of SDA-framework interactions since the synthesis differed only in the ratio of Si and Ge, which both possess the same charge. The synthesis yielded zeolites with **UTL** structure as verified by the X-ray powder diffraction (XRD) (Figure 2a). We calculated the crystallite size along *a-*, *b-,* and *c*-axes from the full width at half-maximum (fwhm) of 200, 020, and 001 diffraction peaks at 2θ = 6.12, 12.64, and 7.36°, respectively. The overall crystallite size increased with increasing Si/Ge ratio. The lower germanium content decelerated the nucleation resulting in longer crystallization time and the formation of larger crystals. Additionally, the *h*/*l* and *k*/*l* aspect ratios of the crystallites also increased with Si/Ge implying that the crystallite shape also changes with Si/Ge (Figure 2b). Particularly, the crystallites became thicker and wider along the *a-* and *b*-axes with increasing content of Si. It is important to note that the crystallite size is not interchangeable with the bulk crystal morphology of the **UTL** (as the bulk crystallites can consist of a large number of small crystallite domains) and does not have to follow the same trends.

**Figure 3:**
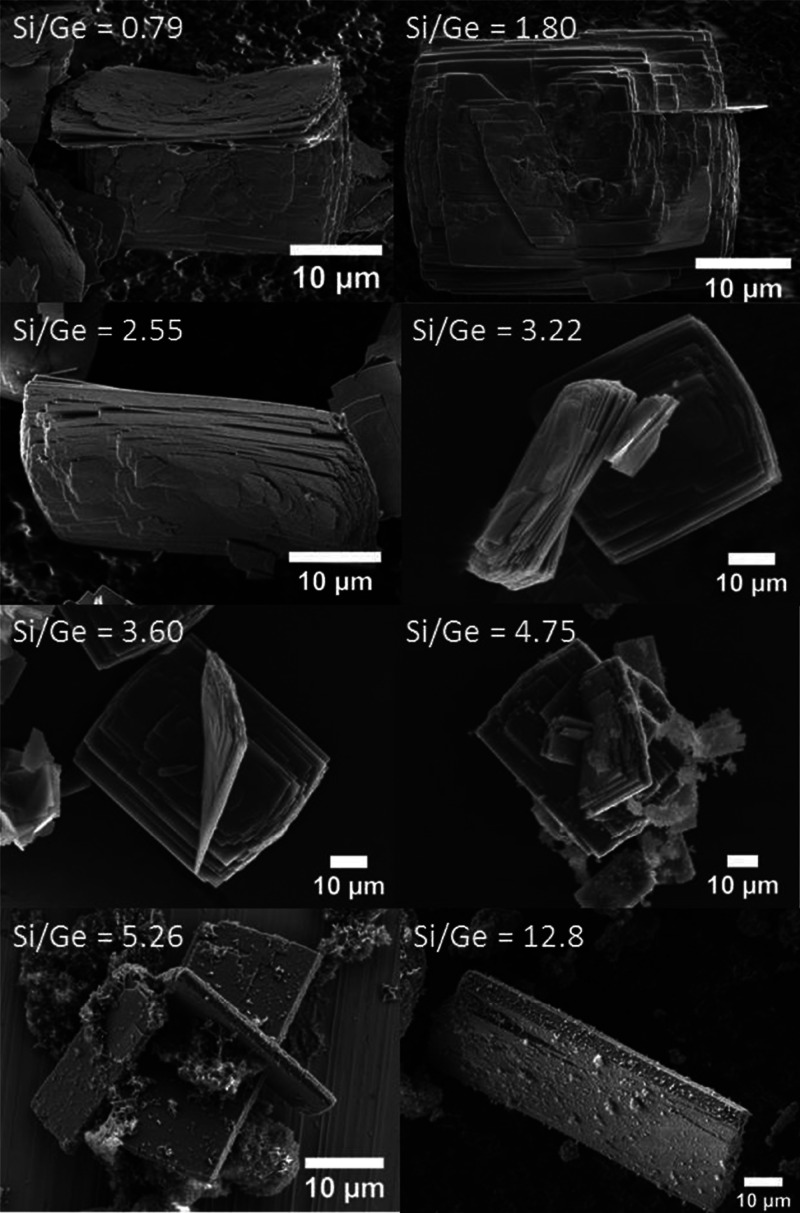
SEM images of **UTL** zeolites with varying Si/Ge molar ratios.

Hence, we analyzed the bulk morphology of the **UTL** crystals by SEM imaging. The SEM images showed that the **UTL** with Si/Ge = 0.79 consisted of thin rectangular crystals, averaging 30, 23, and 0.16 μm along the *c-*, *b-* and *a-*axis, respectively (Figure 3). The average size was calculated from images of at least five separate crystals.

**Figure 4:**
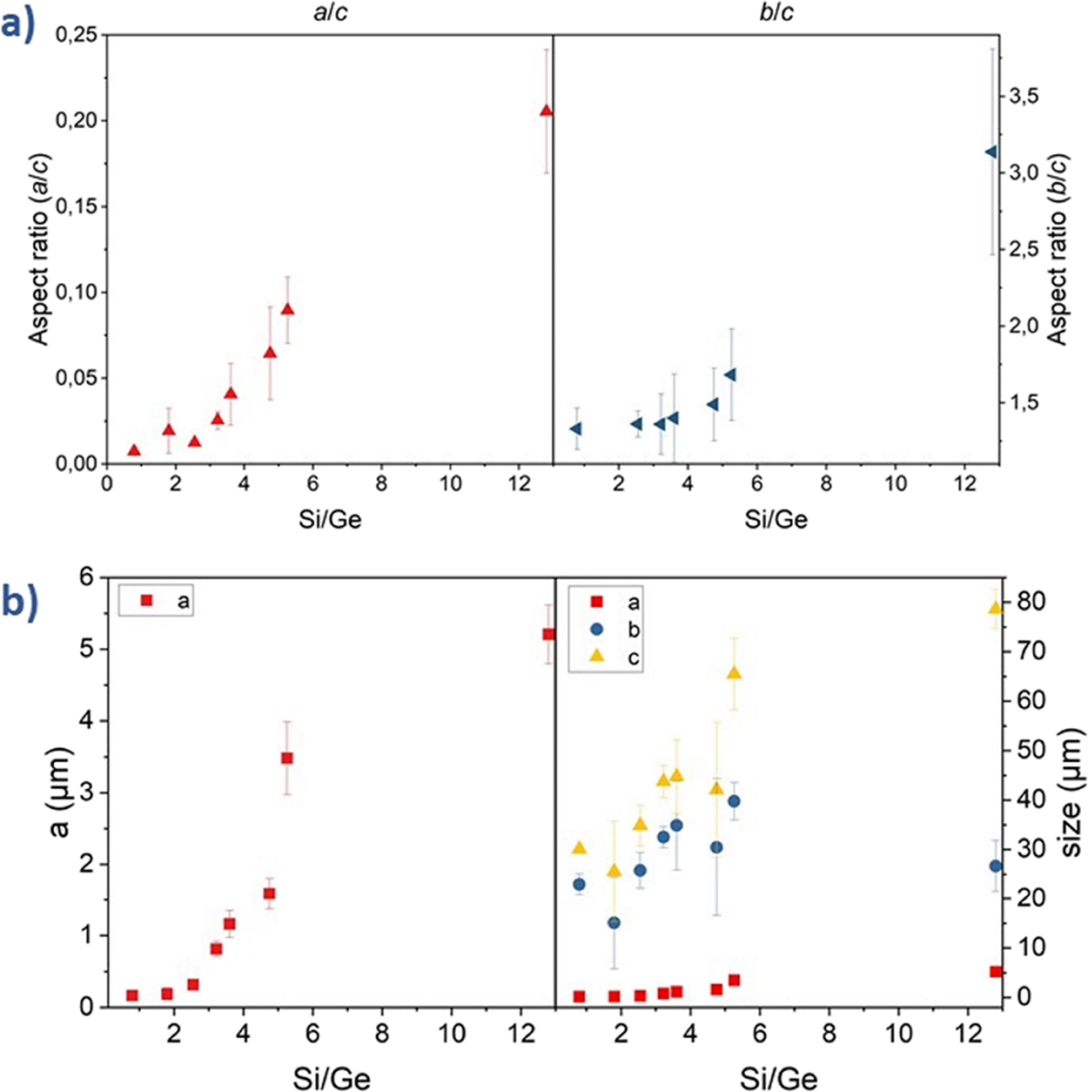
(a) Variation of crystal aspect ratios and (b) variation of crystal sizes along the *a-*, *b-,* and *c-*axes as a function of Si/Ge.

Increasing the Si/Ge ratio from 0.79 to 12.8 progressively increased the average crystal size to 79, 27, and 5.4 μm along the *c-*, *b-,* and *a-*axis, respectively (Figure 4b). To assess the variation of the crystal morphology as a function of Si/Ge, we calculated the crystal aspect ratios along the *b*/*c* and *a*/*c* axes. Figure 4a shows a steady increase in *b*/*c* and *a*/*c* aspect ratios with Si/Ge, highlighting that Si/Ge alone determines the crystal morphology of **UTL** germanosilicate. **UTL** crystals undergo two morphological changes. First, the *b*/*c* aspect ratio increases with the increase in the Si/Ge ratio (i.e., with the decrease in Ge content) because germanium is crucial for the formation of *t-cub* (D4R) units. The density of the *t-cub* units is higher along the *c*-axis. Increasing the germanium content favors the formation of these units and, as a result, the expansion of the crystal along the *c*-axis, which manifests as decreasing *b*/*c* aspect ratio with increasing germanium content.

**UTL** samples with Si/Ge ≥4.75 also contained particles of amorphous matter (Figure 3). This amorphous phase was also inferred from the textural properties of the samples, especially from the low volume of the micropores, more specifically 0.15 and 0.03 cm^3^/g, in samples with Si/Ge = 5.26 and 12.8, respectively (Table S1). The argon adsorption isotherms of these samples also deviate from the typical type I toward type II due to interparticle adsorption at higher relative pressures (Figure S2). The interparticle adsorption likely occurs between the amorphous particles in the sample. The amorphous particles originated from leftover uncrystallized synthesis gel, suggesting that the nucleation rate decreases with the increase in the Si/Ge ratio. If germanium is essential to nucleation in proportion to silicon, then silicon cannot be fully consumed in Ge-poor synthesis mixtures. Consequently, a portion of the unused silica precipitates, forming amorphous particles.

Ge-rich mixtures (samples with Si/Ge = 0.79 and 1.80) contained more germanium than **UTL** can accommodate. Unsurprisingly, the excess germanium precipitated as germanium oxide, thereby decreasing the micropore volume (Figure S3).^9^ The decrease in the nucleation rate with the increase in Si/Ge led to the formation of larger crystals. These results are in line with our observations (Figure 3) and with previous findings of Shvets et al., who also noticed a similar variation in **UTL** zeolite crystal morphology and size as a function of the Si/Ge molar ratio.^39^

Based on the comment of the referee, we have also considered the synthesis duration as a possible factor that may influence the **UTL** crystal morphology. The anisotropic nature of the **UTL** framework implies that the crystal growth rate varies along the individual crystallographic axes. Hence, the crystal shape and aspect ratios may, hypothetically, evolve with time. The characteristic powder XRD pattern of **UTL** emerged after only 3 days of synthesis time (Figure S4a). Nevertheless, sampling of the synthesis mixture in a range from 2 to 13 days revealed no clear evolution of crystal shape in time (Figure S4b). The sample recovered after 3 days contained typical flat crystals 32 × 21 × 0.23 μm^3^ in size covered in smaller amorphous particles (Figure S5). The intensity of the characteristic XRD reflections increased with synthesis time accompanied by the gradual vanishing of the amorphous matter, while the average crystal size and aspect ratio remained virtually unchanged. It is clear, that the **UTL** undergoes rapid crystal growth in the early stage of the synthesis (<2 days) followed by a relatively stagnant stage characterized by only minor changes in crystal size and aspect ratio.

In summary, increasing the Si/Ge ratio decelerates the nucleation and prolongs the crystallization time of the **UTL** germanosilicate. On the other hand, both isolated crystallites and bulk crystals of **UTL** exhibit increase of *a*/*c* and *b*/*c* crystal aspect ratio, meaning that the crystal morphology also changes. The germanium atoms promote the nucleation but at the same time impede the crystal growth along the *a*- and partially along the *b*-axis.

### Extension to Other Frameworks

Similar response to the Si/Al ratio has been observed for the **MWW** zeolite. The **MWW** is an aluminosilicate zeolite with a two-dimensional 10–10R channel system. **MWW** aluminosilicate crystallizes as a semiordered layered precursor MCM-22P, as monolayered (disordered) MCM-56 or as 3D MCM-49.^46,50^ MCM-22P is formed by decreasing the aluminum content in the synthesis gel so that the layers are aligned into a semiordered structure through H-bonding but lack covalent interlayer connections. In contrast, preparing a fully connected three-dimensional MCM-49 framework requires a high aluminum content and involves the formation of a unilamellar intermediate MCM-56 consisting of disordered single layers. Under specific conditions, MCM-56 is the final product.^45,47^ Yet, despite its structural specificities, **MWW** zeolites are formed following patterns similar to those discussed above. The aluminum in the synthesis mixture, on the one hand, facilitates the formation of interlayer connections and a condensed 3D framework, but on the other hand, decelerates crystal growth in the interlayer direction. Additionally, the entire gel converts first to unilamellar MCM-56 and only after to MCM-49 (3D form) under moderately alkaline conditions.^51^ The reduced growth rate of **MWW** results in thinner crystals along the interlayer axis. In the presence of aniline and under high H_2_O/Si ratio, crystal thickness decreases to that of a single unit cell, yielding isolated MCM-56 layers.^46,47^

Crystal morphology is also correlated to elemental composition in other zeolites, as outlined in Table 1. For example, increasing the Si/Al molar ratio (i.e., the silicon content) of **LTL**, with unidimensional 12R pores, promotes growth perpendicularly to the pores, increasing the width and simultaneously decreasing the length of the crystals, which consist of hexagonal rods elongated in the direction of the 12R pores.^22^ Similarly, in **IFR** zeolite, which also contains unidimensional 12R channels, decreasing the content of aluminum from 1.52 Al per unit cell to 0.44 Al per unit cell (i.e., increasing the Si/Al ratio) produces thicker prismatic crystals elongated along the direction of the pores^41^ Decreasing the aluminum content in **MTW** zeolite from Si/Al ratio 35 to 120 also increases the zeolite crystal size by slowing nucleation; however, without significantly affecting the crystal morphology. The morphology change may not have occurred due to the relatively low aluminum content in the respective samples.^42^ In this study, the Si/Ge effect on **UTL** crystal morphology weakened when increasing Si/Ge concentrations to 5.26 and 12.8 (Figure 3b). Moreover, increasing the Si/Ge ratio to 12.8 decreased the overall crystallinity (Figure 3a), promoting the formation of amorphous unused silica (vide infra). Unfortunately, we were unable to synthesize **UTL** at higher Si/Ge concentrations (data not shown). Therefore, we could not verify whether the effect is exclusive for low Si/T ratios, and the morphology of **UTL** remains unaffected at Si/Ge between 35 and 120.

**Table 1:**
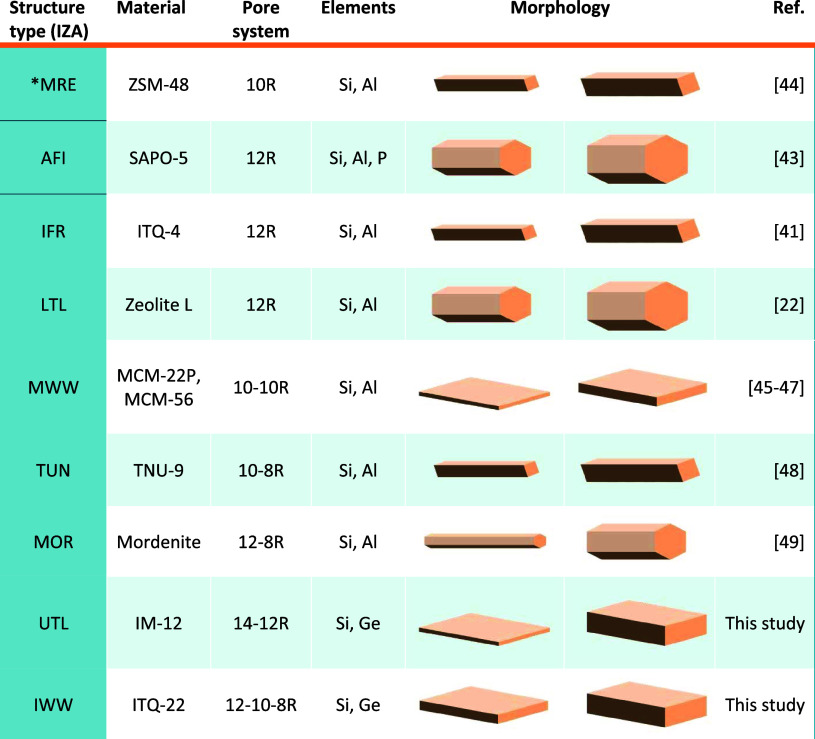
List of Zeolite Structures Whose Crystal Morphology Varies as a Function of the Si/T Ratio

Silico-aluminophosphate SAPO-5 (**AFI**, 12R) undergoes a similar change in morphology transition as a function of its Si content because increasing the Si content produces thicker crystals perpendicularly to the channel system.^43^ This example suggests that the correlation between crystal morphology and Si/T ratio may be valid for a wide range of porous materials beyond zeolites.

Overall, our results with **UTL** zeolite corroborate the findings of studies with other zeolites, summarized in Table 1, which consistently report the correlation between zeolite crystal morphology and its Si/T molar ratio. By extrapolation, we propose that the crystal morphology of zeolites (and possibly other porous solids) with a one- or two-dimensional pore system predictably varies with the Si/T ratio. Increasing the Si content of a framework promotes crystal growth in the crystallographic dimension perpendicular to the direction of the pores, resulting in thicker crystals in the respective direction(s).

### IWW

As shown above and in previous studies,^22,43−49^ increasing Si/T enhances crystal growth in directions perpendicular to the pores in a wide range of zeolites with one- or two-dimensional channel systems with significant structural anisotropy. Such framework anisotropy should also be observed in anisotropic zeolites with 3D channel systems of unevenly large pores. To test this assumption, we selected **IWW** (ITQ-22) germanosilicate as a model zeolite.^52^

**Figure 5:**
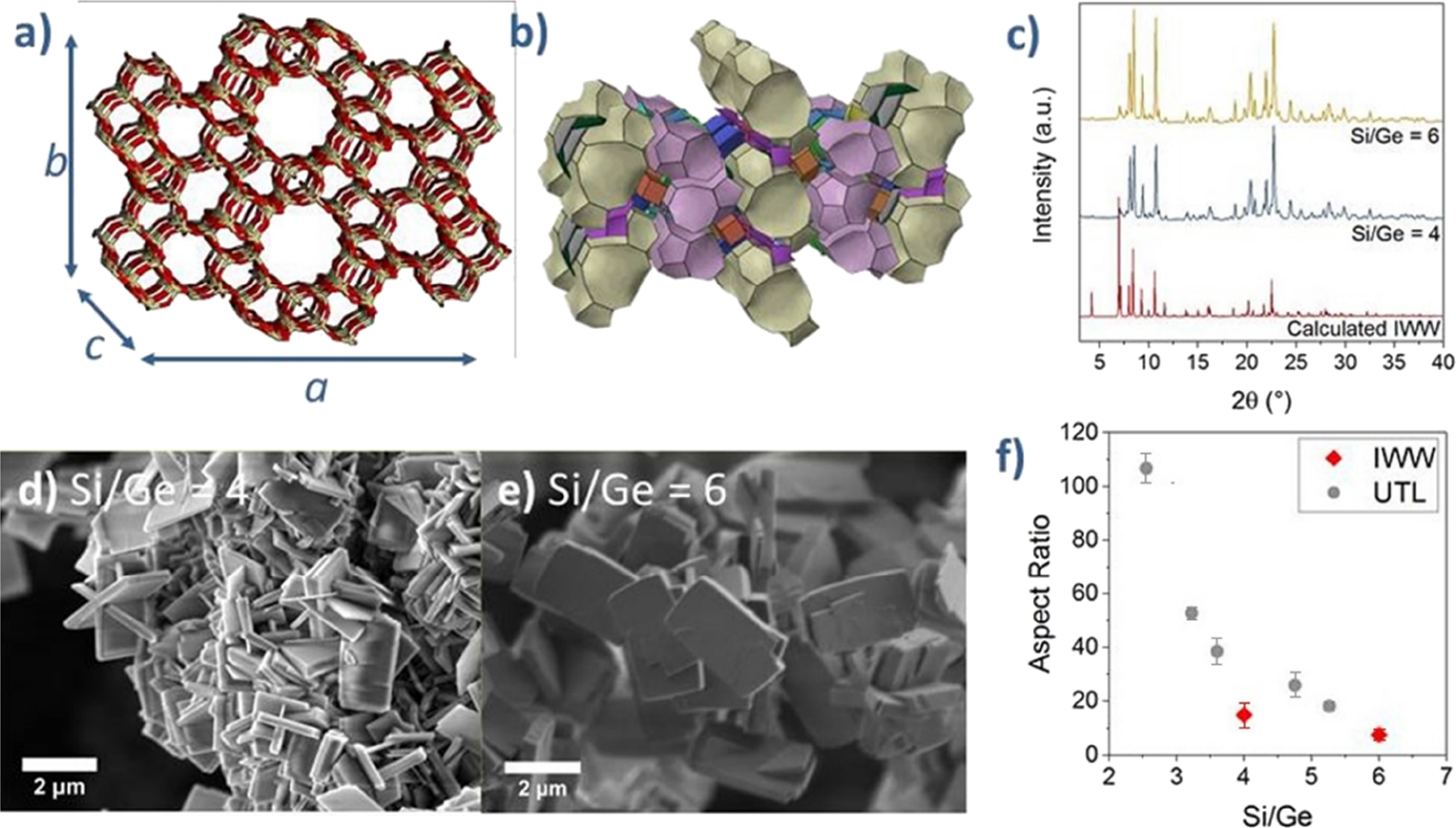
(a) Schematic representation of the **IWW** structure; (b) arrangement of the building units in the **IWW** structure; (c) powder XRD patterns of the **IWW** samples; (d) SEM image of **IWW** (Si/Ge = 4); (e) SEM image of **IWW** (Si/Ge = 6); and (f) variation of the **IWW** and **UTL** crystal aspect ratios as a function of the Si/Ge.

Because the **IWW** structure contains a three-dimensional system of interconnected 12-, 10-, and 8R pores (Figure 5a), **IWW** crystals should be elongated in the directions of the *c*-axis parallel to the largest 12R pores. Considering the arrangement of the large building units into a row along the *b*-axis (Figure 5b), we further hypothesized that the **IWW** crystal is longer along the *b*-axis than along the *a*-axis and grows more along the *b-* and *a-*axes, perpendicular to the 12R channels, when increasing the Si/Ge ratio.

The powder XRD patterns of our **IWW** zeolite samples with Si/Ge = 4 and 6 showed reflections typical of the **IWW** framework (Figure 5c). Nevertheless, we observed differences in their relative intensities, which might have resulted from the preferred crystal orientation, suggesting differences in the crystal morphology between samples. For this reason, we analyzed the crystal morphology of the **IWW** samples by SEM.

SEM imaging of **IWW** germanosilicate with Si/Ge = 4 revealed agglomerates and intergrowths of flat rectangular crystals averaging 2.36 μm × 1.34 μm × 0.16 μm (Figure 5d,e). In turn, transmission electron microscopy (TEM) imaging of the **IWW** sample with Si/Ge = 4 enabled us to identify the crystallographic axes in the IWW zeolite crystals (Figure S6). At high magnification, we confirmed that the projection of the crystal lattice matches the *a,b-*plane of the **IWW** topology. We also verified the crystal *a,b*-facet by comparing our SEM images to those of a previously published HR-TEM study of **IWW**.^53^ Based on the “broken” *a*-edge, which gives the *a,b-*facet its distinguished semihexagonal shape, we identified the crystallographic directions. The crystal sizes were *a* = 0.16 μm, *b* = 2.36 μm, and *c* = 1.34 μm, in line with our estimates; the crystals were elongated along the *c-*axis, while the array of 12R pores decelerated the growth along the *a*-axis, thus reducing crystal size in the *a*-axis. The reason for the unexpectedly pronounced *b*-axis growth remains elusive.

Upon increasing the Si/Ge ratio from 4 to 6, the size of the **IWW** crystals increased from 0.16 × 2.36 × 1.34 to 0.38 × 2.79 × 1.50 μm (Figure 5e). **IWW** showed the most pronounced growth along the *a*-axis, which lies perpendicular to the array of the 12R pores. The aspect ratio of the **IWW** zeolite followed the trend observed in **UTL** (Figure 4f), thereby validating our hypothesis that the morphology of anisotropic zeolites varies with their Si/T ratio whether they have one-, two-, or three-dimensional channel systems.

## Conclusions

The crystal morphology of the **UTL** germanosilicate zeolite predictably varies with the Si/Ge molar ratio. The Si/Ge ratio determines the aspect ratio of the crystallographic *b-* and *c-*axes parallel to the two-dimensional pore system of **UTL** against the perpendicular *a-*axis. This uneven crystal shape reflects differences in crystal growth rates along different axes caused by the uneven arrangement of the building units. In other aluminosilicate and germanosilicate zeolites with anisotropic structural features (e.g., one- or two-dimensional pore system), including **MWW**, **MOR**, *MRE, or **LTL**, increasing the Si/T ratio also promotes crystal growth in direction(s) perpendicular to the pore system or arrays of large building units. Therefore, zeolite crystal morphology can be predicted and controlled based on the Si/T ratio, and this approach is valid for a wide range of zeolites with varying structure topologies and compositions. Because crystal morphology is crucial for tuning the textural and mechanical properties of zeolite-based materials, this approach may be a simple and reliable scale-up method.

## Experimental Methods

### Synthesis of SDAs

**UTL** germanosilicates were synthesized using 2,6-dimethyl-5-azoniaspiro[4.5]decane (DMASD) bromide as a SDA.^39^ DMASD was prepared by mixing 60 mL of 1,4-dibromobutane, 82.9 g of K_2_CO_3_, and 500 mL of acetonitrile in a round-bottom flask. In addition, 67 mL of 2,6-dimethylpiperidine was added to the mixture. The reaction was performed at 85 °C, refluxing for 24 h. Subsequently, the acetonitrile was evaporated. The solid product was dissolved in ethanol, and the insoluble K_2_CO_3_ was removed by filtration. The ethanol solution was concentrated by evaporation; DMASD was precipitated by the addition of diethyl ether and recovered by filtration. The structure of DMASD was verified by ^1^H NMR spectroscopy. This SDA was ion exchanged for the hydroxide form using Ambersep 900(OH) ion-exchange resin with a 2:1 SDA:resin w/w ratio.

**IWW** germanosilicates were synthesized using 1,5-bis(methylpyrrolidinium)pentane dihydroxide (MPP).^52^ MPP was prepared by mixing 18.8 g of 1,5-dibromopentane and 20 g of *N*-methylpyrrolidine in 150 mL of acetone and refluxed for 20 h. The product was collected by filtration, washed with acetone, and dried under vacuum overnight. The structure of the SDA was verified by ^1^H NMR spectroscopy, using deuterium oxide as a solvent. MPP was ion exchanged for hydroxide form using anionic exchange resin (OH type of Ambersep 900) (8 mmol of SDA/g resin). Subsequently, the excess water in the SDA solution was evaporated at 35 Torr and 35 °C to a hydroxide concentration of 1.0 M.

### **UTL** Synthesis

The germanosilicate **UTL** zeolite was prepared by dissolving germanium oxide in a 0.6 M solution (unless stated otherwise) of DMASD hydroxide. Once the germanium oxide was fully dissolved, fumed silica (Cab-O-Sil) was added to the mixture and stirred until dissolved. The final molar composition of the synthesis mixture was *x*SiO_2_:1 – *x*GeO_2_:0.4 SDA: 33.3H_2_O, where *x* represents the Si/(Si + Ge) molar ratio. The pH of the synthesis gel was adjusted to 10.5 by the addition of DMASD hydroxide or diluted (0.01 M) HCl solution. Diluted 0.01 M solution of LiOH was used to adjust the pH of **UTL** synthesis with reduced SDA/Si molar ratios. The synthesis mixture was transferred to 20 mL Teflon-lined stainless-steel autoclaves. Crystallization was performed at 175 °C for 8 days with agitation (200 rpm). The resulting product was recovered by filtration, washed with water, and dried at 60 °C. The products were calcined at 550 °C for 6 h in airflow to remove the organic template.

### **IWW** Synthesis

The germanosilicate **IWW** samples were prepared by dissolving the required amounts of germanium dioxide (Sigma-Aldrich, 99.99%) and tetraethyl orthosilicate (Sigma-Aldrich, 98%) in MPP solution. The ethanol formed by hydrolysis of tetraethylorthosilicate was evaporated under stirring. The final mixture with molar composition *x*SiO_2_:1 – *x*GeO_2_:0.25 MPP:15H_2_O was transferred to Teflon-lined stainless-steel autoclaves and heated to 175 °C K for 11 days. The final products were recovered by centrifugation, washed with water, and dried at 60 °C overnight. The resulting solids were calcined at 580 °C for 6 h in air.

### Characterization Methods

The crystallinity and crystal structure of the samples were analyzed by XRD on a Bruker D8 Advance diffractometer equipped with a Linxeye XE-T detector in the Bragg–Brentano geometry using Cu Kα (λ = 0.15406 nm) radiation. Data were collected over the 2θ range of 3–40° with a 0.021° step size at 0.8 s per step. Crystallite size, *L*, in *h*00, 0*k*0, and 00*l* directions was calculated using the Scherrer equation:
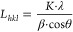
where *K* represents the shape factor fixed at 0.89, λ is the wavelength of the X-ray source, β is the fwhm of the respective diffraction peak, and θ is its diffraction angle.

Crystal size and morphology were examined by SEM imaging under a JEOL IT-200 microscope in secondary electron imaging mode at an electron beam accelerating voltage of 15 kV and a working distance of 10 mm. Additional measurements were performed using a JEOL IT-800 microscope in secondary electron imaging mode at an electron beam accelerating voltage of 3 kV and a working distance of 2 mm. Aspect ratios of the crystals were calculated as *x*_1_ divided by *x*_2_, where *x*_1_ and *x*_1_ represent two of the crystallographic axes. The presented aspect ratio values were calculated as an arithmetic average of values obtained from at least five different crystals:
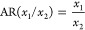
The Ge content and Si/Ge ratio of zeolites were determined by inductively coupled plasma mass spectrometry (ICP-MS) analysis (Agilent 7900 ICP-MS; Agilent Technologies, Inc., USA). Approximately 50 mg of the sample was mixed with 1.8 mL of HNO_3_ (67–69%, ANALPURE), 5.4 mL of HCl (34–37%, ANALPURE), 1.8 mL of HF (47–51%, ANALPURE), and then transferred into a closed Teflon vessel, placed in the microwave (Speedwave XPERT, Berghof) and heated at 210 °C (5 °C/min) for 25 min. After cooling, the complexation of the surplus HF was performed by adding 12 mL of H_3_BO_3_, followed by microwave treatment at 190 °C (5 °C/min) for 10 min. Once cooled down, the solutions were diluted for analysis.

TEM imaging was performed using a JEOL NeoARM 200 F microscope equipped with a Schottky-type field emission gun operated at an accelerating voltage of 200 kV. The microscope was aligned using a gold nanoparticle sample as the standard to reach atomic resolution.

Argon adsorption–desorption isotherms were obtained on a Micromeritics 3Flex volumetric Surface Area Analyzer at −186 °C in liquid argon bath. The samples were degassed on a Micromeritics Smart Vac Prep instrument under vacuum at 250 °C for 8 h with heating rate 1 °C min^–1^ under vacuum (3 × 10^–2^ mmHg minimum pressure). The specific surface area was calculated using the Brunauer–Emmett–Teller method in the relative pressure range from *p*/*p*_0_ = 0.05 to *p*/*p*_0_ = 0.25. The micropore volume (*V*_mic_) was calculated by using the *t*-plot method. The total pore volume (*V*_tot_) was calculated from the adsorbed amount at a relative pressure of *p*/*p*_0_ = 0.98.

## Supporting Information Available

SEM images of **UTL** zeolite crystal with spatial *a-*, *b*-, and *c*-axes; argon adsorption–desorption isotherms, and textural properties of **UTL** samples; correlation of the Si/Ge molar ratios in the synthesis gel and in solid and sample pore volumes; and powder XRD patterns and SEM images of **UTL** samples with varying synthesis time (PDF)

## Footnotes

1 In this study, we adopted the lowercase, 3-letter designation of layers according to Gies and Marler^34^ Marler, B.; Gies, H. Hydrous layer silicates as precursors for zeolites obtained through topotactic condensation: a review, Eur. J. Mineral. **2012**, *24*, 405–428.
