# Supplementary material for: Controlling Crystal Morphology of Anisotropic Zeolites with Elemental Composition
Source: Cryst Growth Des. 2024 Mar 8;24(6):2406–14. doi: 10.1021/acs.cgd.3c01312 (PMC10958493; doi:10.1021/acs.cgd.3c01312)
Supplement: Supplementary file 1 — cg3c01312_si_001.pdf [file cg3c01312_si_001.pdf]

Supplementary Information

# Controlling crystal morphology of anisotropic zeolites with elemental composition

Ondřej Veselý[1], Mariya Shamzhy[1], Wiesław J. Roth[2], Russell E. Morris[3], and Jiří Čejka[1]

[1]*Faculty of Sciences, Charles University, Hlavova 8, 128 43 Prague 2, Czech Republic*
[2]*Jagiellonian University, Faculty of Chemistry, Gronostajowa 2, 30-387 Krakow, Poland*
[3]*EaStChem School of Chemistry, University of St. Andrews, North Haugh, St. Andrews, Fife, KY16 9ST, UK*

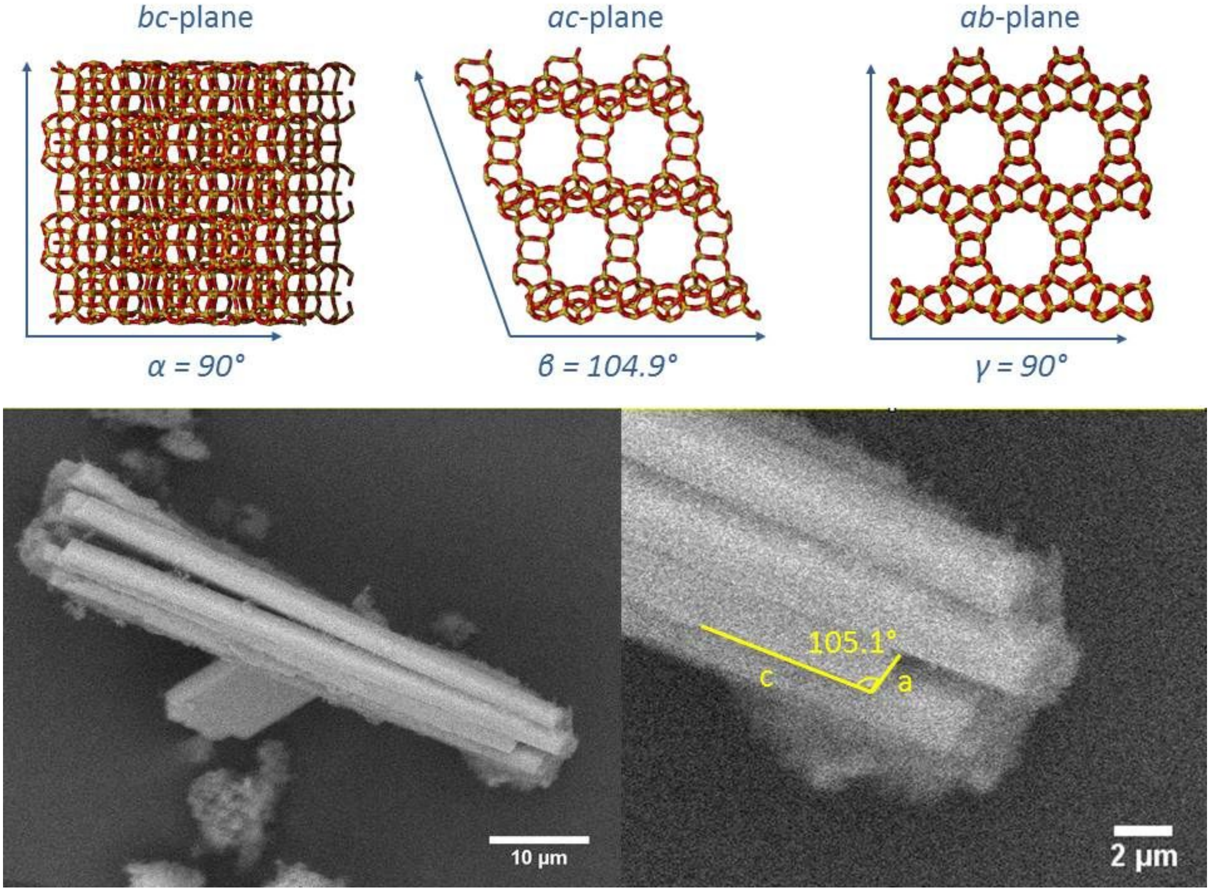


Figure S1: Schematic representation of the **UTL** zeolite crystal with projections along the a-, b- and c-axis and SEM image of the **UTL** (Si/Ge = 5.26) sample with the highlighted crystallographic plane angle corresponding to the geometry of the **UTL** topology

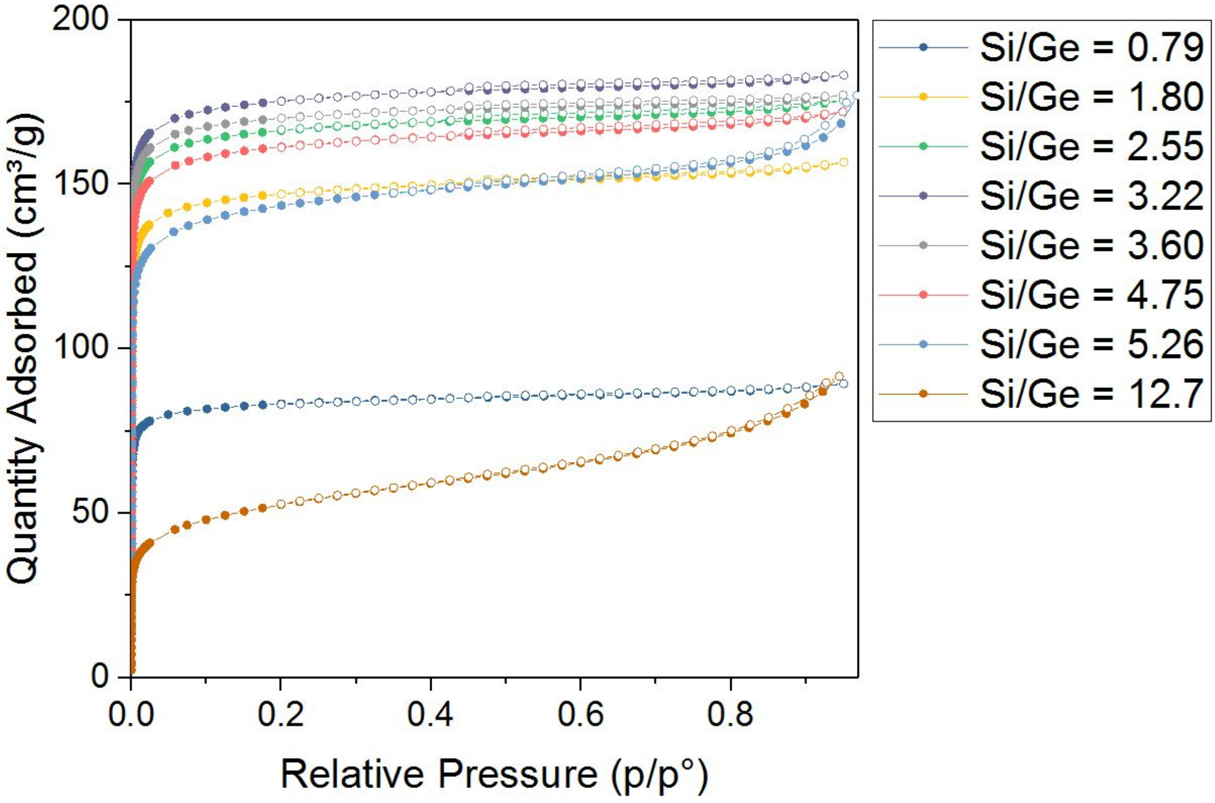


Figure S2: Argon adsorption-desorption isotherms of **UTL** germanosilicate samples

Table S1: Textural properties of **UTL** germanosilicate samples determined by argon adsorption

| Si/Ge (synthesis) | Si/Ge (product) | BET ($m^2/g$) | $S_{external}$ ($m^2/g$) | $V_{total}$ ($cm^3/g$) | $V_{micro}$ ($cm^3/g$) |
|---|---|---|---|---|---|
| 0.33 | 0.79 | 250 | 8.6 | 0.11 | 0.10 |
| 0.50 | 1.80 | 442 | 15 | 0.20 | 0.17 |
| 1.00 | 2.55 | 494 | 42 | 0.22 | 0.19 |
| 2.00 | 3.22 | 520 | 42 | 0.23 | 0.21 |
| 3.00 | 3.6 | 505 | 38 | 0.23 | 0.20 |
| 4.00 | 4.75 | 480 | 45 | 0.22 | 0.20 |
| 5.00 | 5.26 | 430 | 66 | 0.23 | 0.15 |
| 7.00 | 12.8 | 162 | 49 | 0.12 | 0.03 |

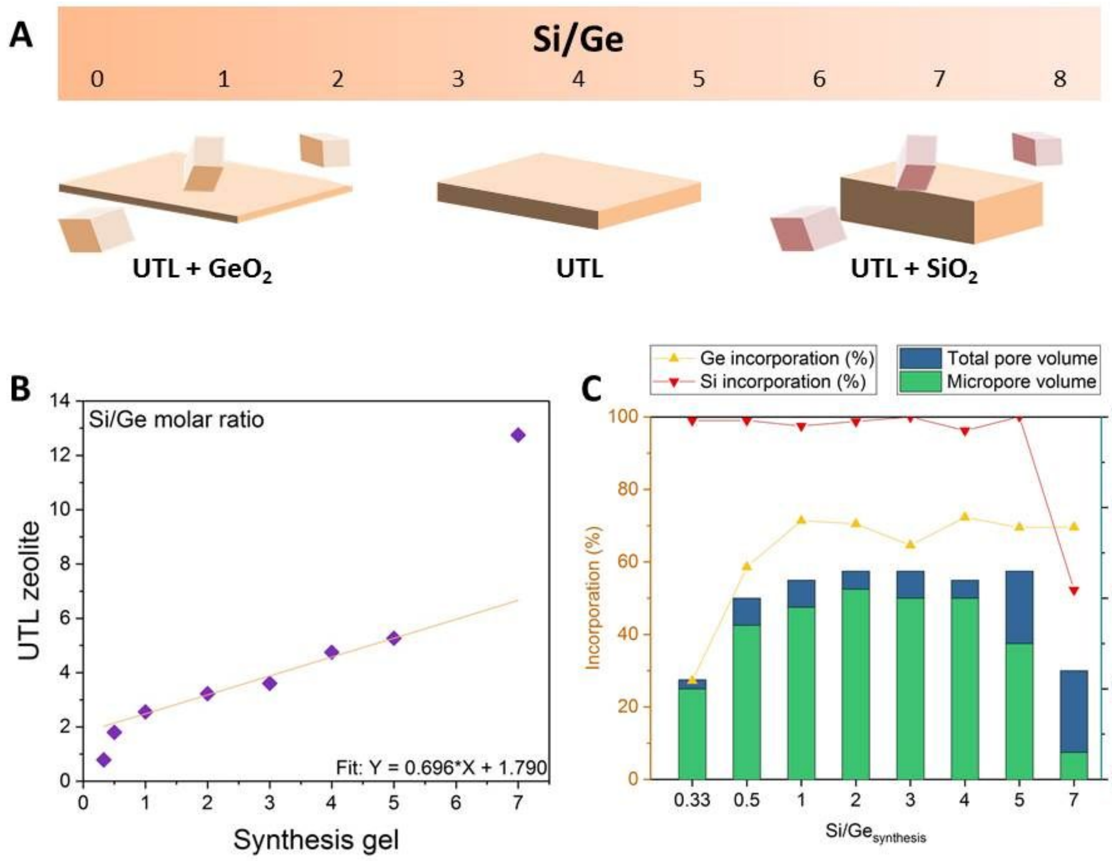


Figure S3: A) Schematic illustration of $SiO_2$/$GeO_2$ precipitation; B) correlation of the Si/Ge molar ration in the synthesis gel and in the recovered bulk solid; C) correlation of the Si and Ge incorporation (%) with sample total pore volumes and micropore volumes.

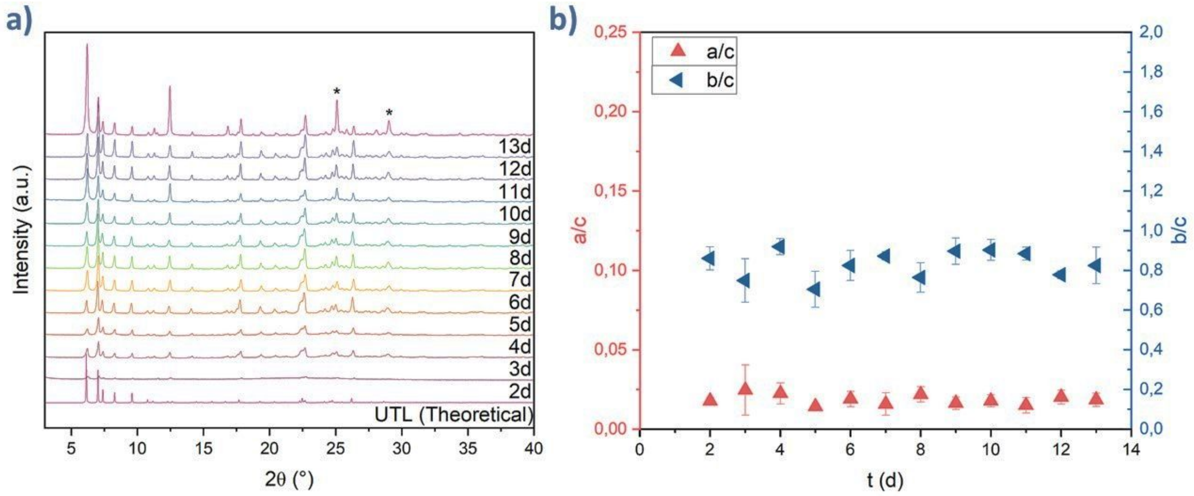


Figure S4: a) powder XRD patterns of **UTL** samples (* = reflections of the product of re-crystalisation) and b) variation of crystal aspect ratios as a function of time.

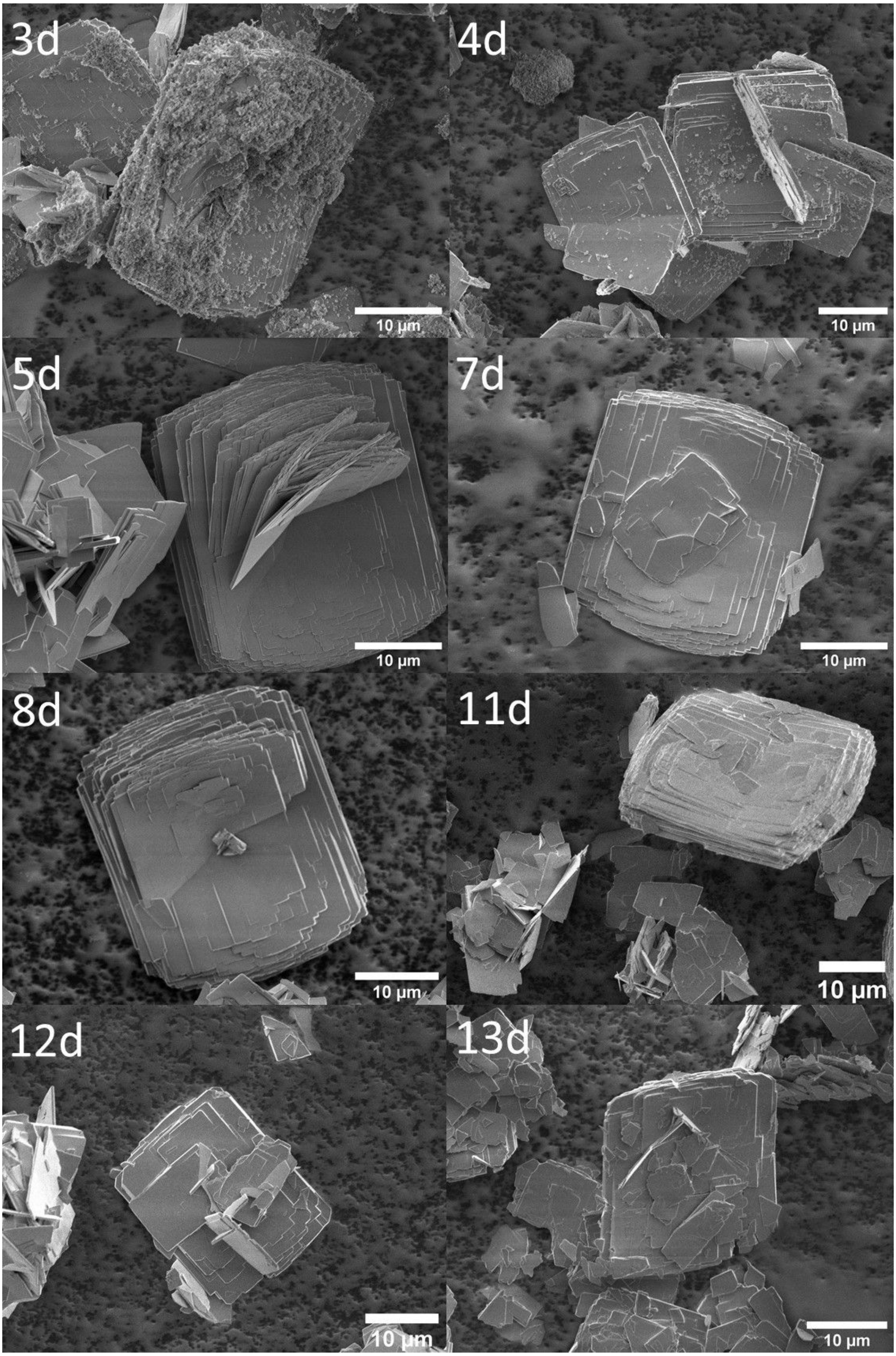


Figure S5: SEM images of **UTL** zeolite samples recovered in different synthesis durations.

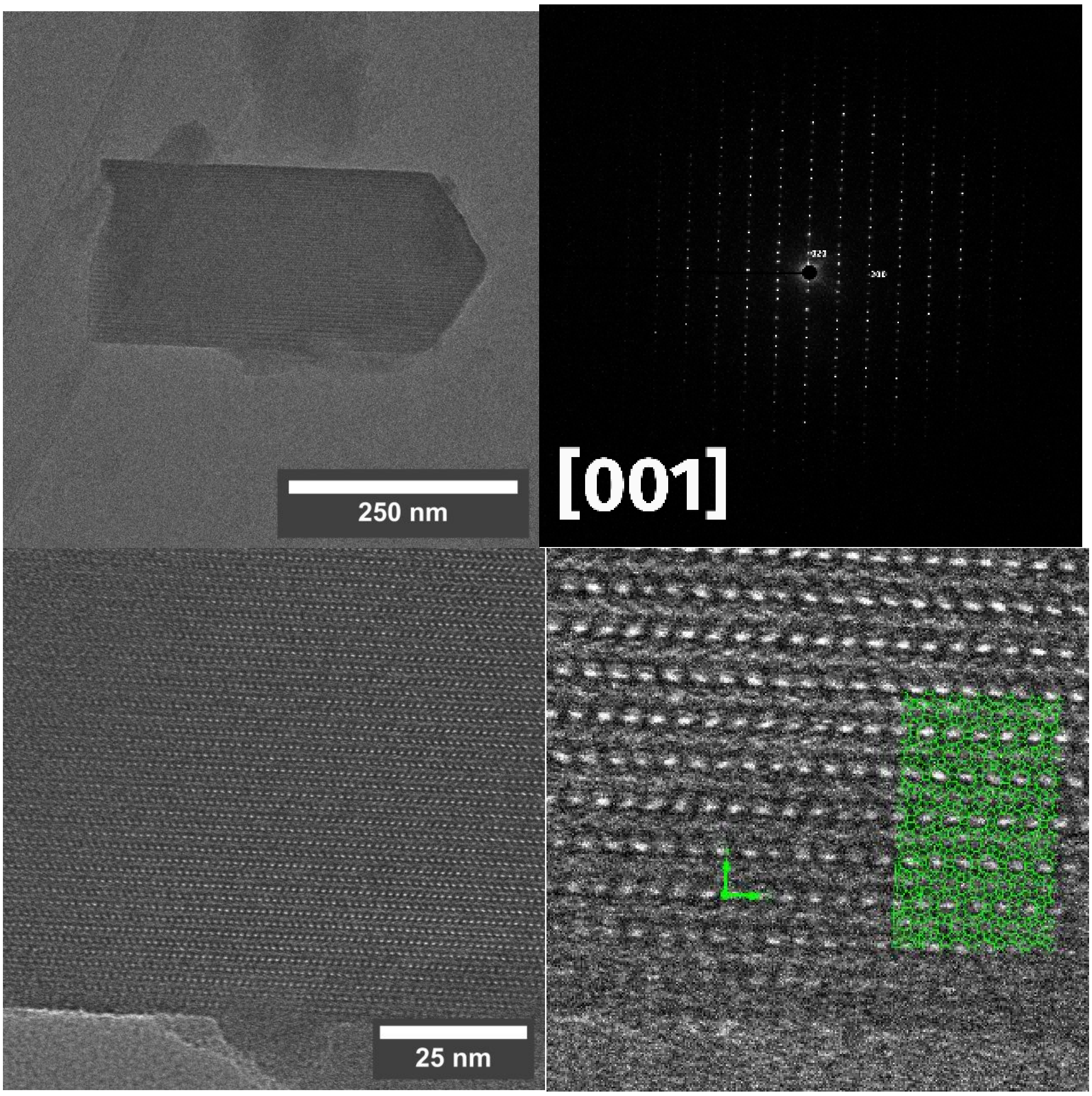


Figure S6: TEM images and electron diffraction of the **IWW** (Si/Ge = 4) crystal compared to the theoretical **IWW** topology and crystallographic axes
